# Spatially Guided Construction of Multilayered Epidermal Models Recapturing Structural Hierarchy and Cell–Cell Junctions

**DOI:** 10.1002/smsc.202200051

**Published:** 2022-09-14

**Authors:** Haiwei Zhai, Xiaowei Jin, Grayson Minnick, Jordan Rosenbohm, Mohammed Abdul Haleem Hafiz, Ruiguo Yang, Fanben Meng

**Affiliations:** ^1^ Department of Mechanical and Materials Engineering University of Nebraska-Lincoln Lincoln NE 68588 USA; ^2^ Nebraska Center for Integrated Biomolecular Communication University of Nebraska-Lincoln Lincoln NE 68588 USA

**Keywords:** 3D bioprinting, cell junctions, cell self-organization, collective migration, in vitro skin models, keratinocyte differentiation, pemphigus

## Abstract

A current challenge in 3D bioprinting of skin equivalents is to recreate the distinct basal and suprabasal layers and promote their direct interactions. Such a structural arrangement is essential to establish 3D stratified epidermis disease models, such as for the autoimmune skin disease pemphigus vulgaris (PV), which targets the cell–cell junctions at the interface of the basal and suprabasal layers. Inspired by epithelial regeneration in wound healing, a method that combines 3D bioprinting and spatially guided self‐reorganization of keratinocytes is developed to recapture the fine structural hierarchy that lies in the deep layers of the epidermis. Herein, keratinocyte‐laden fibrin hydrogels are bioprinted to create geographical cues, guiding dynamic self‐reorganization of cells through collective migration, keratinocyte differentiation, and vertical expansion. This process results in a region of self‐organized multilayers (SOMs) that contain the basal‐to‐suprabasal transition, marked by the expressed levels of different types of keratins that indicate differentiation. Finally, the reconstructed skin tissue as an in vitro platform to study the pathogenic effects of PV is demonstrated, illuminating a significant difference in cell–cell junction dissociation induced by PV antibodies in different epidermis layers, which indicates their applications in the preclinical test of possible therapies.

## Graphical abstract

A stepwise biofabrication method that combines 3D bioprinting and postprint cell self‐organization is developed for epidermal reconstruction. Skin‐cell‐laden tissue architectures are created to display the multilayered structures seen in vivo. These 3D models recapitulate the basal‐to‐suprabasal transition, providing an in vitro tool to mimic the pathological microenvironment of the disease pemphigus vulgaris.

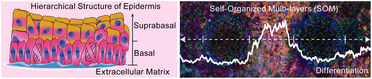

## 1 Introduction

Cell–cell adhesions play an essential role in numerous physiological processes by maintaining the paracellular barrier, establishing intercellular communication and tuning mechanical strength.^[^ ^1^ ^]^ Disorders of cell–cell adhesions are associated with various diseases.^[^ ^2^, ^3^ ^]^ For example, the skin blistering disease pemphigus is caused by epithelial cell junction disruption.^[^ ^4^ ^]^ In the most common and potentially fatal form of pemphigus, pemphigus vulgaris (PV), autoantibodies attack desmosomes in keratinocytes, primarily the desmosomal cadherin desmoglein 3 (Dsg3), leading to the disruption of cell–cell adhesion at the basal and suprabasal layers, as well as their interface.^[^ ^5^ ^]^ Studies have shown that the immunological perspective is insufficient in understanding the mechanism of the disease and guiding therapeutic support.^[^ ^6^, ^7^, ^8^ ^]^ There is strong evidence that the microenvironments of cell–cell adhesions significantly contribute, and potentially dictate, the pathogenesis of PV.^[^ ^9^ ^]^ However, most of these studies were conducted using 2D keratinocyte monolayers. Though convenient, these monolayers often fail to reproduce critical in vivo characteristics of the epidermis, such as the structural hierarchy, the geometrical complexity, and the cell–extracellular matrix (ECM) interactions.^[^ ^10^ ^]^ Hence, there is a need to construct in vitro 3D stratified tissue architectures that better mimic physiological and pathological microenvironments, allowing for increased precision in the prediction of cellular behaviors during disease progression and in response to potential therapies.

Indeed, 3D‐cultured keratinocytes have been shown to behave differently from those cultured in a monolayer, especially in response to inflammation and mechanical stimulation that causes alterations of cell–cell and cell–ECM interactions.^[^ ^10^, ^11^ ^]^ Current efforts to produce stratified epithelium by 3D bioprinting have introduced desired matrices and recreated tissue architectures in a layer‐by‐layer fashion.^[^ ^12^, ^13^ ^]^ However, limited by resolutions of current 3D printing technologies, they are unable to generate a stratified structural equivalent which mimics the fine layers at basal and suprabasal locations. These deep epidermal tissue layers are often a few tens of micrometers in thickness, consisting of less than ten layers of cells,^[^ ^14^ ^]^ while a cell–matrix combination with a thickness of ≈100 μm is usually deposited for each layer during 3D bioprinting.^[^ ^15^ ^]^ Of interest, the basal and suprabasal layers are the targets of PV antibodies, where the antibody‐induced cell–cell adhesion disruption originates.^[^ ^16^ ^]^ The direct cell–cell interactions across these layers are also crucial in the pathogenesis of blistering in PV.^[^ ^17^, ^18^ ^]^ In vitro recapitulation of such compact cell–cell adhesions in a 3D arrangement is still a major challenge for scaffold‐based biofabrication methods, as the degradation rate of the supporting matrices is typically required to match the pace of new tissue formation.^[^ ^19^ ^]^ Although skin organoids produced by stem cell differentiation and self‐organization could be possible solutions, these self‐assembled miniorgans often do not allow precise spatial control.^[^ ^20^ ^]^

Combining 3D bioprinting and self‐guided cell organization seen in skin wound healing, we seek to build a multilayered epidermal model to recreate the nuanced structural arrangement of the basal‐to‐suprabasal transition. We aim to use this 3D skin tissue construct as a model system to study the pathogenesis of PV in defined microenvironments. By taking advantage of 3D bioprinting, sources of keratinocytes are precisely placed to create a spatially defined interspace, providing geographical cues to guide cell activities post fabrication. This biofabrication strategy results in the formation of a self‐organized multilayer (SOM) of keratinocytes via collective migration and self‐triggered differentiation, which can restore the structural hierarchy of the basal and suprabasal layers.^[^ ^1^ ^]^ Finally, toward disease modeling, we demonstrate that PV antibodies induce desmosome disassembly and disrupt cell–cell adhesions in this SOM‐based skin model. Furthermore, the cell–cell junctions from each epidermal layer exhibit significant differences in response to the antibody treatment. Our bioengineered skin model may inspire a shift in current 3D skin bioprinting practices to facilitate self‐guided reconstruction of keratinocytes.

## 2 Results and Discussion

**Scheme 1:**
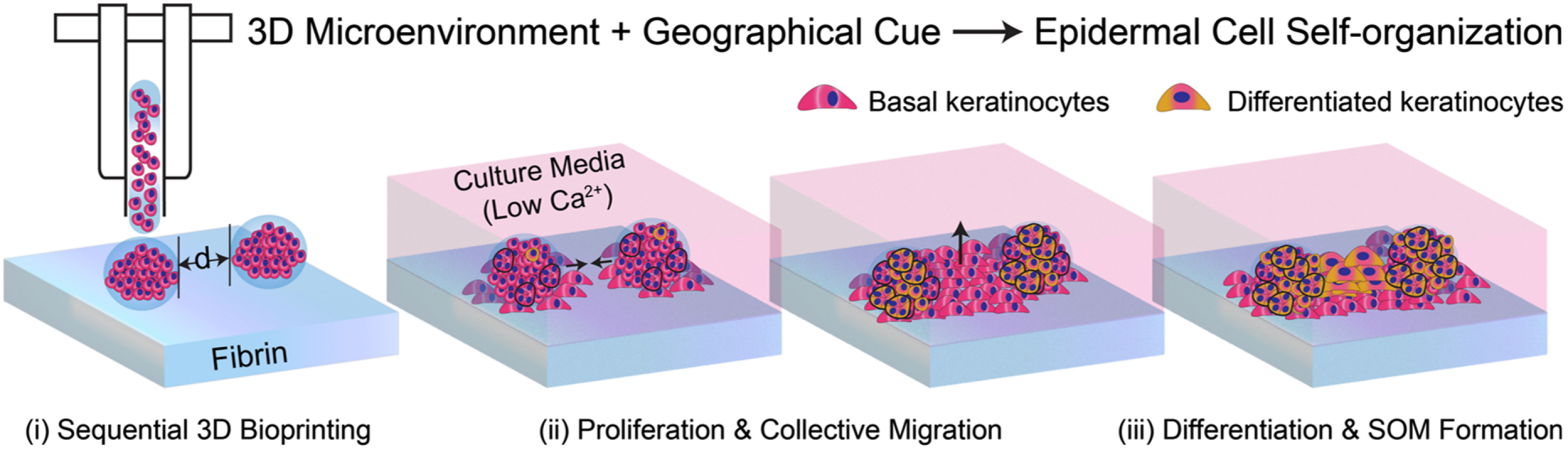
Spatially guided construction of stratified epidermal models. Schematic image of the workflow of epidermal model fabrication. (i) Keratinocyte‐laden fibrin droplets are bioprinted as initial cell sources and geographical cues. (ii) 3D‐cultured cells proliferate and collectively migrate to generate a confluent basal layer between cell source droplets. (iii) The defined interspace results in high cell density triggering basal‐to‐suprabasal differentiation and multilayer formation.

The construction of multilayered epidermal tissues combines 3D bioprinting and guided cell self‐organization with the conceptual design illustrated in **Scheme** **1**. Inspired by epidermis regeneration during wound healing, optimized epidermal bioinks are sequentially deposited using a custom‐built 3D printer with a spatial control over cell sources. The chemical composition, cell‐loading density, and biodegradability of the bioprinted constructs are finely tuned to provide a 3D microenvironment promoting rapid proliferation and collective migration of keratinocytes. The interspace (*d*) between cell sources is precisely defined to create geographical cues guiding the formation of compact basal layers and the ensuant basal‐to‐suprabasal differentiation, which leads to the regeneration of hierarchical epidermal architectures.

### 2.1 3D Keratinocyte Culture through Fibrin Encapsulation

To fundamentally understand essential cell activities including keratinocyte proliferation, migration, and differentiation in 3D microenvironments, we used a conventional hydrogel‐scaffold method to build a culture platform for keratinocytes. Here, HaCaT cells, a widely used human keratinocyte line, were encapsulated in a 3D hydrogel matrix. The HaCaT cells were engineered to express green fluorescent protein (GFP)‐tagged E‐Cadherin (GFP‐E‐cad) for real‐time imaging. Natural fibrin was selected as the scaffold material, owing to its well‐known biocompatibility and biodegradability.^[^ ^21^, ^22^, ^23^, ^24^ ^]^ Fibrin is also an essential material of hemostatic plugs to assist skin regeneration during wound healing.^[^ ^25^, ^26^, ^27^, ^28^, ^29^ ^]^ In a typical experiment, 1 × 10^6^ cells mL^−1^ of HaCaT cells were first encapsulated in a 1.5 μL fibrin hydrogel at a concentration of 10 mg mL^−1^. Then, the cell‐laden droplet was placed on a bulk‐supporting fibrin hydrogel (≈150 μL). The proliferation and migration of encapsulated keratinocytes from the spatially defined primary site were subsequently tracked and characterized.

**Figure 1:**
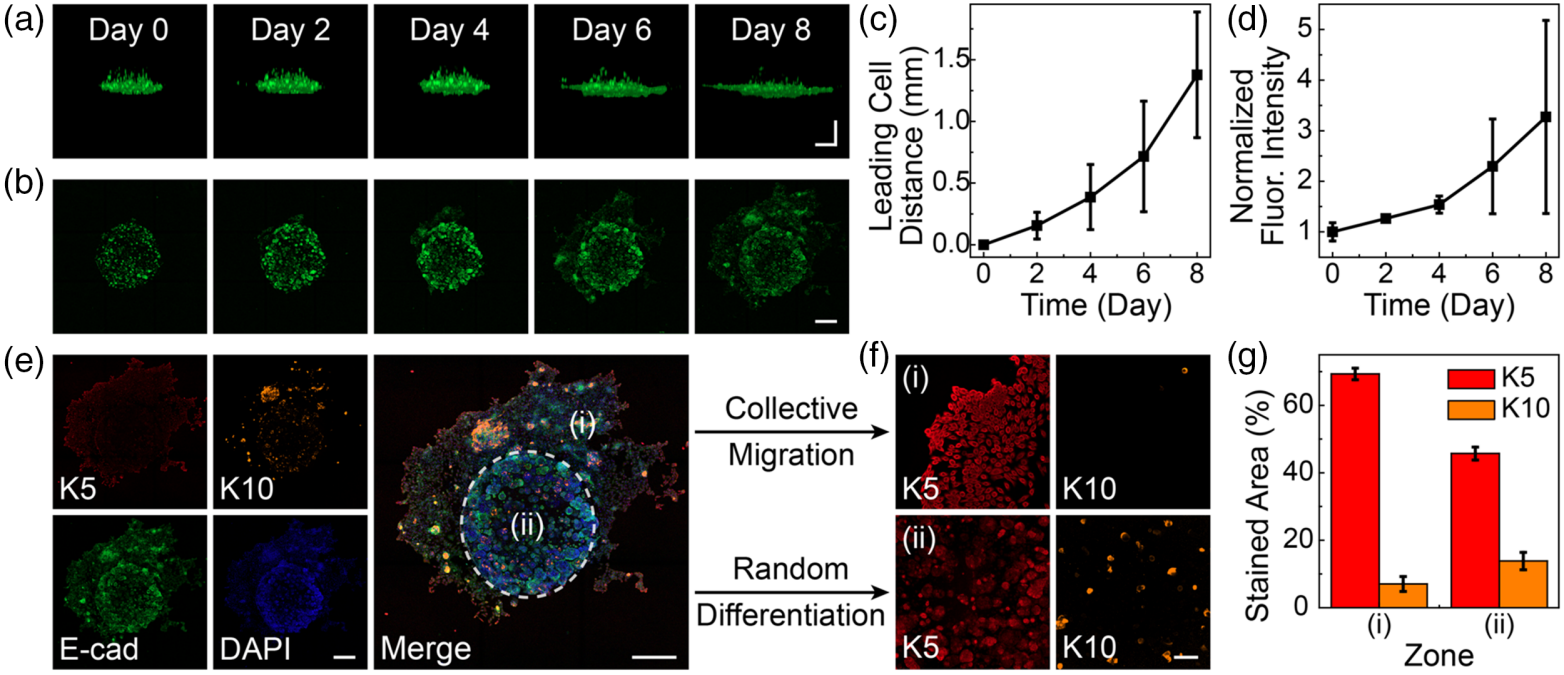
3D‐cultured keratinocytes with fibrin encapsulation. a,b) Time‐lapse *Z*‐stacked confocal images showing the distribution of GFP‐E‐cad‐HaCaT cells from the side (a) and the top‐down (b) view over time. Scale bar: 500 μm. c) Plots of the distance of leading HaCaT cells versus time, demonstrating the cell migration (mean ± s.d., *n* ≥ 4). d) Plots of cellular fluorescence intensity of HaCaT cells versus time, demonstrating the cell proliferation (normalized by intensity at day 0, mean ± s.d., *n* ≥ 4). e) Panoramic *Z*‐stacked fluorescence images showing the collective migration of 3D‐cultured HaCaT cells in the surrounding fibrin gel matrix, zone (i), from the cell source droplet, zone (ii), and the self‐triggered differentiation that randomly occurred in the biofabricated construct. (Red: K5, Orange: K10, Green: GFP‐E‐cad, and Blue: DAPI). Scale bar: 500 μm. f) Representative fluorescence images showing HaCaT cell distribution and differentiation status. Scale bar: 100 μm. g) Bar charts showing the expressions of K5 and K10 in zones (i) and (ii) (mean ± s.d., *n* = 3).

Cellular activities were monitored by capturing time‐lapse fluorescence images with confocal microscopy. **Figure** **1**a,b shows the distribution of HaCaT cells over an 8‐day period after cells adapted to the 3D hydrogel matrix. From the comparison of cell distribution at each imaging day, two main features of cellular activities were observed, namely, spatial expansion and increased cell population. Both cell migration (Figure 1c) and proliferation (Figure 1d) were quantified by analyzing fluorescence intensity of GFP‐E‐cad expressed by keratinocytes, which provided experimental guidance for the design of multilayered epidermal models in the following steps. As shown in Figure 1b,c, cells migrated through the surrounding 3D matrix and out from the primary seeding droplet. The leading cells could travel ≈1.4 mm away from the boundary of droplets on day 8. In the meantime, summed GFP‐E‐cad intensity exhibited greater than threefold enhancement (Figure 1d), indicating significant cell population increase. It should be noted that the gel formula was optimized to maintain the 3D environment over the test window and to facilitate the activities of encapsulated cells (Figure S1, Supporting Information). In addition, aprotinin, an antifibrinolytic protein, was added into the fibrin matrix to tune its degradation rate, promoting collective migration of keratinocytes in the surrounding fibrin matrix (Figure S2, Supporting Information).^[^ ^30^ ^]^

Keratinocyte differentiation is an essential process that drives epidermal stratification and maturation.^[^ ^31^ ^]^ To monitor cellular differentiation, the 3D‐cultured keratinocytes were characterized by immunostaining of key differentiation markers. The panoramic fluorescence images in Figure 1e show a representative 3D‐cultured sample after staining. The green channel represents E‐cad expression, and the blue channel represents cell distribution via conventional DAPI nucleus staining. The red and orange signals are generated from the immunostaining of intermediate filament proteins, keratin 5 (K5) and keratin 10 (K10), respectively. K5 is abundant in basal keratinocytes. Once a basal keratinocyte differentiates and migrates toward the suprabasal layer, K10s are expressed, which help form a new biomechanical environment.^[^ ^32^ ^]^ Therefore, these two keratins can be used to characterize different stages of keratinocyte differentiation. Interestingly, we found a heterogeneous distribution of K5 and K10 over the entire 3D‐cultured sample. As illustrated in Figure 1e, we observed two zones with distinctive features. As highlighted in Figure 1f and S3, Supporting Information, zone (i) was formed by the collective migration of keratinocytes that originated from the deposited fibrin droplets. Cells in zone (i) were continuously packed in a monolayer showing a typical epithelial pattern,^[^ ^33^ ^]^ as observed in the zoom‐in view of the GFP‐E‐cad channel (Figure S4a, Supporting Information), and dominantly expressed K5, indicating their basal status. These collectively migrated cells could serve as the base for the ensuing epidermal regeneration. Compared with zone (i), keratinocytes spontaneously aggregated to numerous cell clusters (Figure S4b, Supporting Information) within the initial droplet (zone (ii)), and meanwhile a higher density of K10 expression (Panel (ii) in Figure 1f) was observed, suggesting the occurrence of keratinocyte differentiation in some cell clusters that were embedded in the 3D matrix. Although differentiated cells are randomly distributed in the droplets, which could not be used to directly mimic the highly hierarchical structure of the epidermis, the cell‐laden droplet did provide sufficient active basal keratinocytes in the 3D microenvironment for the regeneration of a basal layer through collective cell migration. This regional difference was further confirmed by comparing K5‐ and K10‐stained areas in the two zones (Figure 1g).

### 2.2 Dynamic Regeneration of Multilayered Epidermal Models

**Figure 2:**
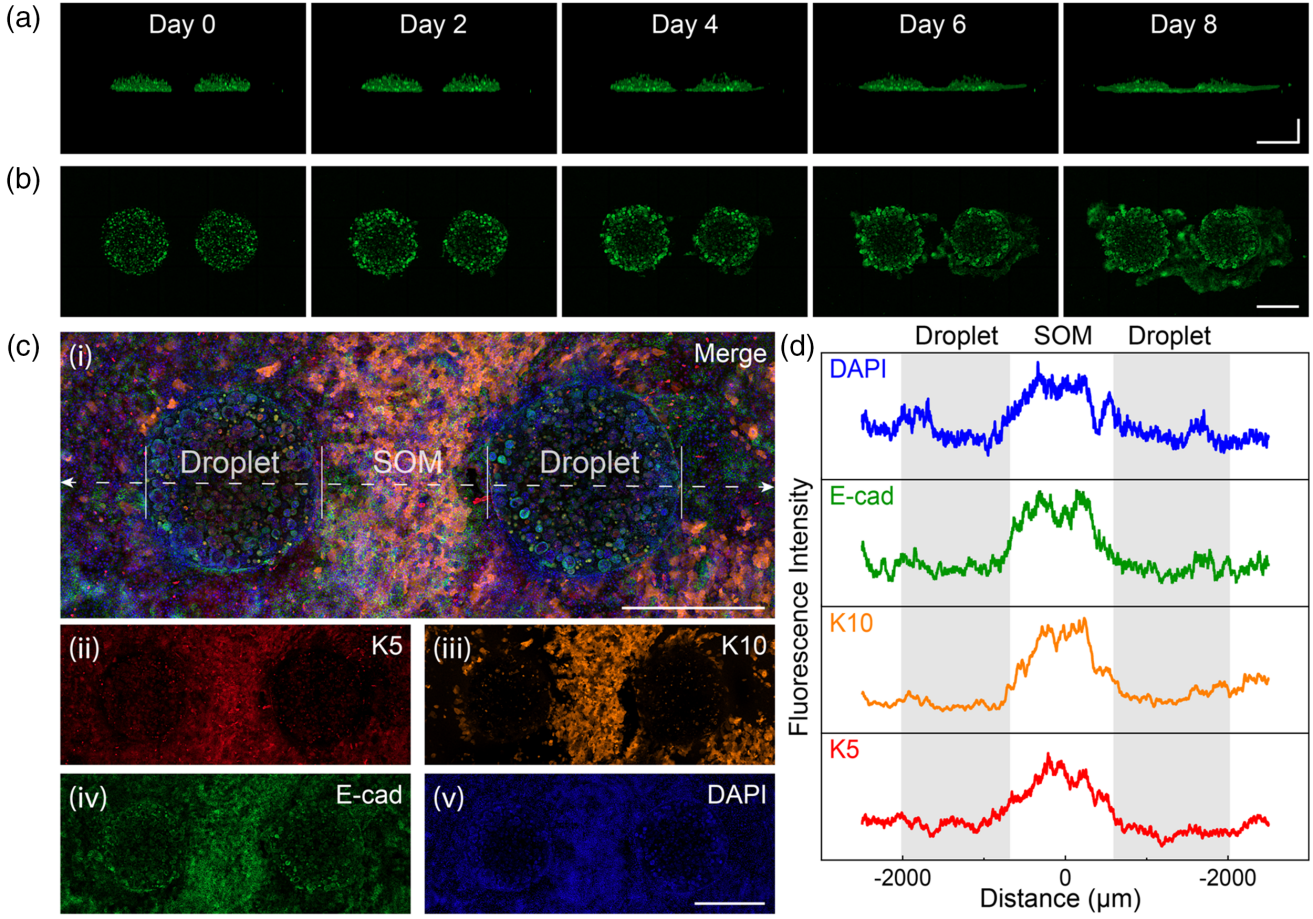
SOM formation at an interspace created between bioprinted cell source droplets. a,b) Time‐lapse panoramic *Z*‐stacked confocal images showing the distribution of GFP‐E‐cad‐HaCaT cells from the side (a) and the top‐down (b) view over time. Scale bar: 1000 μm. c) Panoramic *Z*‐stacked fluorescence images showing spatial distribution of biomarkers and dyes, demonstrating differentiation status of HaCaT cells. (Red: K5, Orange: K10, Green: GFP‐E‐cad, and Blue: DAPI). Scale bar: 1000 μm. d) Plots of fluorescence intensity measured at each pixel along the central axis of a pair of cell source droplets as illustrated by the dashed lines in (c–i).

Inspired by the regeneration of skin barriers during wound healing, we developed a biofabrication strategy by combining 3D bioprinting of cell‐laden fibrin droplets as initial cell sources and postprint cell self‐organization to construct multilayered epidermal tissue, as depicted in Scheme 1. Based on the results discussed in the previous section, two droplets (1.5 μL) of HaCaT cell‐seeded fibrin hydrogel were precisely placed onto a bulk 3D fibrin matrix using a custom‐built 3D bioprinter.^[^ ^34^, ^35^, ^36^ ^]^ It has been established that collective cell activities are influenced by the positioning of cell aggregates.^[^ ^37^, ^38^ ^]^ Therefore, the interdroplet distance (*d*) was selected by taking account of both temporal and spatial efficiency of model construction. As shown in **Figure** **2**a,b, the edge‐to‐edge distance was set between 800 and 1000 μm, which was determined by the observed cell migration rate through the fibrin matrix to allow a 2‐week test window and a roughly 1 mm^2^ area for observation of cell behaviors in response to the subsequent treatments. It should be highlighted that extremely low numbers of cells (≈3 × 10^3^) were used for this fabrication approach, which is more favorable for future applications in precision medicine due to the limited availability of patient cells. Time‐lapse fluorescence images in Figure 2a,b show the proliferation and collective migration of encapsulated keratinocytes. These two processes are critical cell activities for re‐epithelization during skin wound healing.^[^ ^39^ ^]^ With a longer culture time, cells that migrated from the two droplets merged to form a confluent cell monolayer in the defined interspace. Because of the spatial constraint, these tightly packed cells sought to expand vertically, inducing the SOM, similar to the keratinocyte transformation observed during in vivo epidermal stratification.^[^ ^40^ ^]^

Samples were then fixed and stained to evaluate cell differentiation status, as shown in Figure 2c and S5, Supporting Information. Moreover, the spatial distribution of each imaged biomarker was mapped along the central axis of the two keratinocyte‐seeded droplets, as illustrated in Figure 2c, by quantifying fluorescence intensity. Both panoramic microscope images (Figure 2c) and fluorescence intensity plots of four channels (Figure 2d) show the spatial organization of the 3D tissue constructs. The interspace between the two printed droplets exhibited the highest cell density, as multilayered cell architecture was generated via collective migration of encapsulated HaCaT cells (Movie S1, Supporting Information). The strong expression of keratins implied the active re‐epithelization through keratinocyte proliferation (red K5) and differentiation (orange K10, Figure S6, Supporting Information, showing the zoom‐in views) in this region. It also hinted that the tightly packed basal keratinocytes self‐initiated differentiation and drove the formation of epidermal SOMs without creating air–liquid interface^[^ ^41^ ^]^ or introducing external Ca^2+^ stimulation.^[^ ^42^ ^]^ The observed crowding‐triggered keratinocyte differentiation was consistent with previous reports.^[^ ^32^, ^43^ ^]^ Therefore, this dynamic self‐organization of keratinocytes that was guided by the constructed geographical environment could be employed to simulate the formation of skin barriers in vitro.

**Figure 3:**
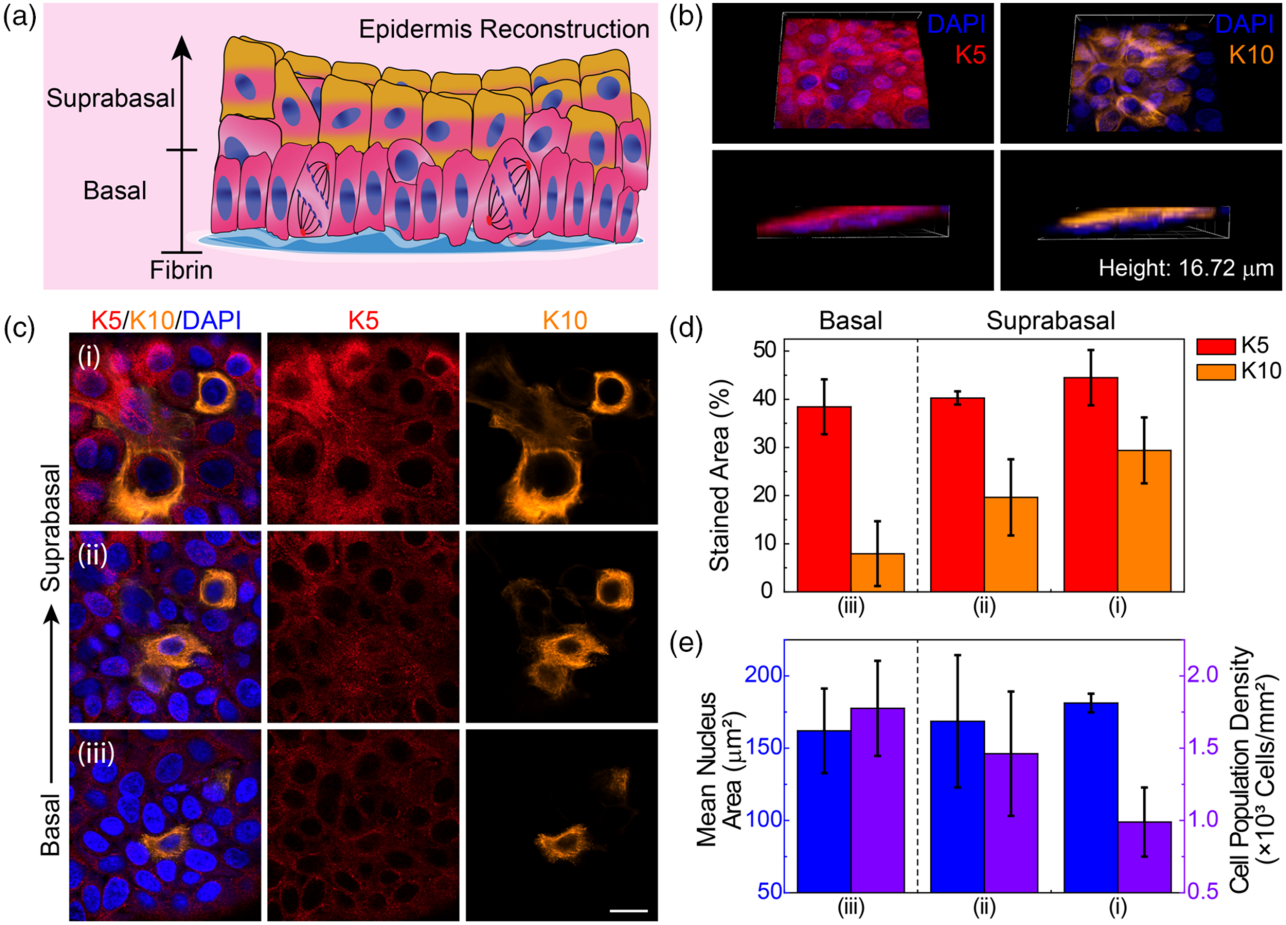
Basal‐to‐suprabasal transition in SOMs. a) Schematic image illustrating the transition of differentiation statuses from basal to suprabasal layers in the SOMs. Red: K5 expressing cells; orange: K10 expressing cells. b) 3D‐reconstructed confocal images showing the spatial distribution of K5 expressing cells (red) and K10 expressing cells (orange) in a scanned volume of 101.41 × 101.41 × 16.72 μm. The lateral views (bottom row) demonstrate the stratification of keratinocyte layers. c) Confocal images of K5/K10/DAPI‐stained HaCaT cells located at three representative layers of an SOM in a bottom‐up scan (five slices with a *Z*‐step of 2.19 μm), demonstrating a vertical transition of cell differentiation statuses. Scale bar: 20 μm. d) Bar charts showing K5 (red) and K10 (orange) stained area of each scanned layer in SOMs, demonstrating the differentiation statuses of HaCaT cells (mean ± s.d., *n* = 3). e) Bar charts showing the mean nucleus area (blue) and the population density (purple) of keratinocytes located in each scanned layer in SOMs, presenting the transition of cellular patterns (mean ± s.d., *n* = 3).

A unique characteristic of the epidermis is its highly hierarchical layer‐by‐layer cell structure. This structural hierarchy is formed by vertical expansion of keratinocytes initiated at the basal layer.^[^ ^31^ ^]^ Through asymmetric mitosis, daughter cells of highly proliferative basal keratinocytes migrate toward the suprabasal layers, differentiate to more rigid cells for the protection function of skin, and simultaneously lose their proliferative capability (illustrated in **Figure** **3**a).^[^ ^44^ ^]^ To highlight the 3D arrangement of keratinocytes and structural hierarchy in these SOMs, representative regions were scanned with a confocal microscope (Figure 3b and Movie S2, Supporting Information). From DAPI signals, it was observed that cells widely distributed in multiple vertical layers. Similar to previous experiments, we stained intermediate filament proteins, K5 and K10. As shown in the lateral views of the bottom‐up scan (Figure 3b, bottom row and Figure S7a, Supporting Information), K10 expression that indicates suprabasal status was mainly detected in top layers with a thickness of 6.58 ± 2.32 μm (turquoise box chart in Figure S7b, Supporting Information) while cells that only expressed K5 were located in the bottom sections (4.44 ± 1.64 μm, red box chart in Figure S7b, Supporting Information), corresponding to basal layers. The total thickness of the SOMs was measured in the range of 5.84–16.72 μm from 14 scanned regions of three independent samples. The construction of such fine stratified epidermal structures is beyond the capability of conventional 3D bioprinting technologies.

To further investigate differentiation stages of keratinocytes in each layer, we selectively captured fluorescence images of three representative horizontal cross sections following the basal‐to‐suprabasal direction (Figure 3c, Movie S3, Supporting Information showing the full vertical scan). In contrast to the random spatial distribution of cell differentiation shown in fibrin droplets, K10 expression was steadily enhanced from the bottom basal layer to the upper suprabasal layers (Figure 3b bottom left panel and Figure 3d, orange bars). This gradual vertical alteration of the differentiation status in SOMs recaptured the process of bottom‐up expansion of basal keratinocytes in vivo.^[^ ^31^ ^]^ Notably, high K5 expression was observed in all keratinocyte layers in the model (Figure 3b bottom right panel and Figure 3d, red bars), which is associated with the proliferative capability of epidermal cells. The coexistence of K5 and K10, especially at upper suprabasal layers, could be due to the immortal nature of the HaCaT cell line that was selected in this study. It is also possible that some differentiated keratinocytes have not completed the epidermal morphogenesis.^[^ ^45^ ^]^

In addition to keratin expression, cell morphology also exhibited a basal‐to‐suprabasal transition within the SOMs of fabricated epidermal models, as shown in Figure 3c. We quantified nucleus size and cell density by calculating the DAPI‐stained area and counting population in each keratinocyte layer, to show this bottom‐to‐top transition of cellular patterns (Figure 3e). We examined the three representative layers in SOMs. With the scan moving upward, the mean nucleus area increased by ≈20 μm^2^ from the basal to upper suprabasal layer whereas the population density declined over 45%. Both results could correspond to enlarged cell areas at a horizontal cross section of 3D tissue constructs. These variations in cell morphology match physiological transitions in layers of the epidermis.^[^ ^31^ ^]^ It has been well established that keratinocytes undergo programmed structural changes during terminal differentiation, and when keratinocytes move up, the upregulated expression of K10 strengthens the cytoskeleton, resulting in cell flattening.^[^ ^5^, ^46^ ^]^ Collectively, these results provide solid evidence that SOMs based on 3D‐cultured keratinocytes could closely mimic the basal‐to‐suprabasal transition of the epidermis at both phenotypic and genotypic levels. The reconstructed skin tissue architectures show the potential as an in vitro platform for epidermal disease models, such as in the study of PV pathology.

### 2.3 Biomimicking the Pathological Microenvironment of PV

We next introduced cell–cell junction disruptions and used these 3D‐fabricated epidermal tissues as a skin disease model. Specifically, to reconstruct the pathological microenvironment of PV in vitro, anti‐Dsg3 antibody (AK23) was added to dissociate the cell–cell junctions by targeting desmosomes in the basal and suprabasal layers. It has been well established that this antibody treatment can induce PV phenotype in both 2D monolayer cultured keratinocytes and animal models.^[^ ^47^, ^48^ ^]^ Compared with these previously reported models, our 3D epidermal architectures were designed to provide a deeper insight regarding spatial arrangements and keratinocyte differentiation status.

**Figure 4:**
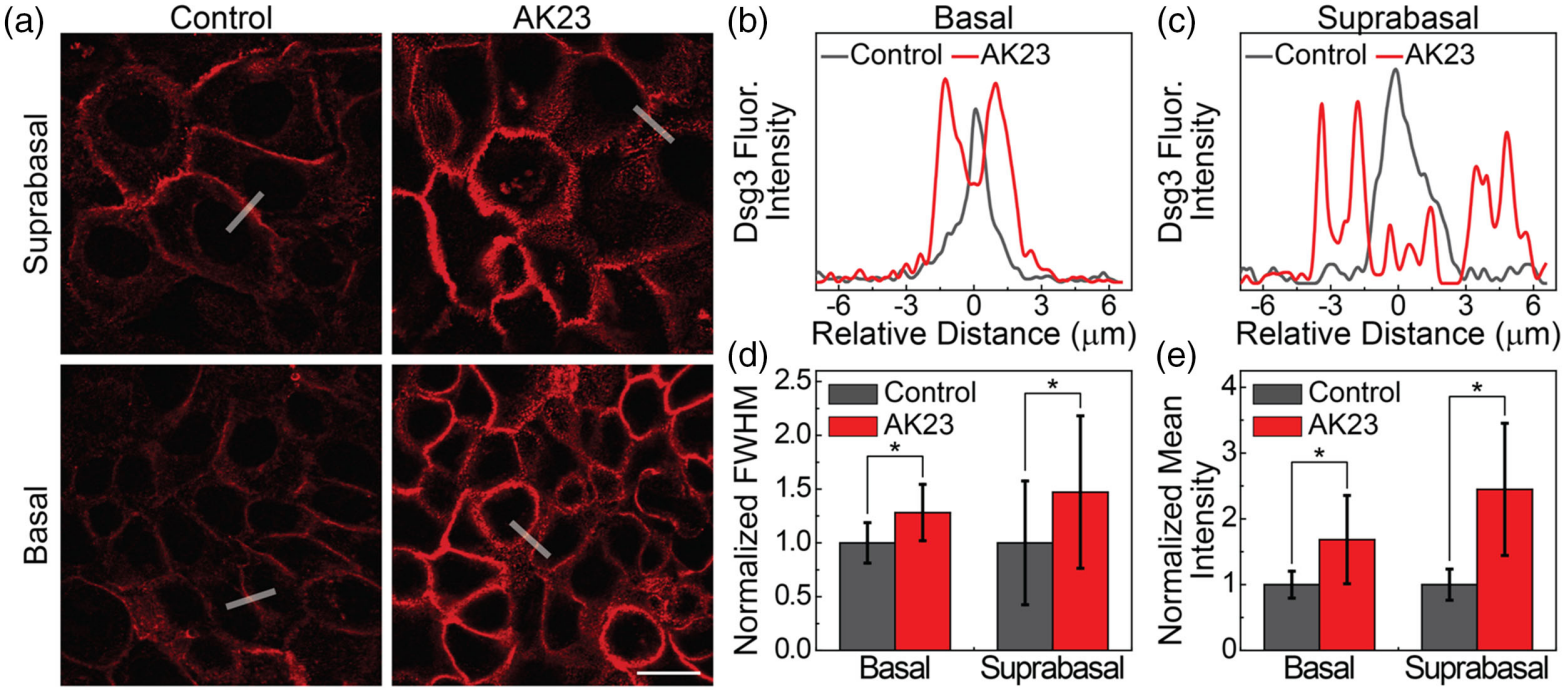
Pathological changes of desmosomal junctions in response to anti‐Dsg3 antibody. a) Confocal images of Dsg3‐stained HaCaT cells at a bottom basal layer and an upper suprabasal layer of representative SOMs without (left column) and with (right column) 2 μg mL^−1^ AK23 mAbs treatment. b,c) Plots of Dsg3 fluorescence intensity of each pixel along a reference line versus relative distance to the line center for a representative desmosomal junction at a bottom basal layer (b) and an upper suprabasal layer (c) of a typical SOM with (red) and without (gray) AK23 treatment. d) Bar charts showing FWHM of representative regions at the basal layer and the suprabasal layer of SOMs with (red) and without (gray) AK23 treatment (normalized by intensity of untreated control group, mean ± s.d., *n* ≥ 7, *p < 0.05). FWHM value was associated with junction width. e) Bar charts showing the mean Dsg3 fluorescence intensity of representative regions at the basal layer and the suprabasal layer of SOMs with (red) and without (gray) AK23 treatment (normalized by intensity of untreated control group, mean ± s.d., *n* ≥ 7, *p < 0.05).

As shown in **Figure** **4**a, Dsg3, the main target of AK23, was labeled by a red fluorescent dye to visualize desmosomal junctions between keratinocytes in the layered epidermis model. In the untreated samples (Figure 4a left column), sharp and clear boundaries were observed, indicating the generation of abundant ordered desmosomes. In contrast, wider and incompact junctions were observed in the AK23‐treated samples, indicating desmosome disassembly (Figure 4a right column). To better analyze desmosome disassembly, cell–cell adhesions were quantitatively characterized by the distribution of Dsg3 fluorescence intensity across randomly sampled junctions, as indicated by white lines in Figure 4a. As shown in Figure 4b,c, the single‐peaked black curves of untreated control samples corresponded to intact cell–cell junctions with Dsg3 that tightly concentrated at the boundary of two adjacent cells, while a series of discrete peaks (red curves) that represent the scattered distribution of Dsg3 were observed at both basal and suprabasal layers after AK23 treatment, which confirmed the dissociation of these desmosomal cadherins. The result of AK23‐induced desmosome disassembly is consistent with previous studies on either 2D keratinocyte monolayers or patient‐derived histological samples.^[^ ^9^, ^47^, ^49^ ^]^ Intriguingly, we found two distinct alterations of cell–cell junctions at the two epidermal cell layers. In a representative AK23‐treated sample, the main Dsg3 peak split into two peaks with a peak‐to‐peak distance of around 3 μm in the basal layer, whereas a more discrete distribution was observed with a distance range of ≈10 μm at the disrupted junction site in the suprabasal layer. Accompanying the wider separation, Dsg3 intensity in the suprabasal layer decreased between each peak at the dissociated junction, suggesting a more thorough dissociation and potentially a complete loss of cohesion between keratinocytes in comparison with the basal layer.

To reinforce the diverse pathological responses of keratinocytes at basal and suprabasal layers, we also compared their full width at half maximum (FWHM) (Figure S8, Supporting Information), which represents the mean width of cell–cell junctions. Both layers showed increased junction width after AK23 treatment as compared with controls (Figure 4d). In addition, a greater degree of variation in junction width was detected in the suprabasal layer than the basal layer, indicating a more aggressive process of junction disruption from AK23 antibody treatment. This difference in junction dissociation caused by physiological locations was further confirmed by the significant increase of overall Dsg3 intensity from basal to suprabasal layers (Figure 4e). This could be attributed to the higher permeability of the looser suprabasal layer, making it more favorable to the immunostaining process. Although the junctional dissociations of keratinocytes have been demonstrated by multiple experimental models, the distinct cellular responses between basal and suprabasal layers have not been reported yet. Therefore, the epidermal models may serve as an in vitro tool to investigate cell behaviors within hierarchical structures of multilayered keratinocytes during the pathogenesis of PV or other skin diseases based on disorders of cell junctions.

## 3 Conclusion

In this study, we developed a new 3D biofabrication approach for the creation of multilayered skin tissue models with epidermal stratification, aiming to establish an in vitro platform that can mimic native physiological and pathological microenvironments. These 3D keratinocyte‐laden architectures were constructed stepwise by combining bioprinting and postprint dynamic self‐arrangement of cells. Structurally, the SOMs in the predefined interspace captured the primary characteristics of basal‐to‐suprabasal hierarchy. Functionally, it was demonstrated that the pathogenesis of PV can be modeled by introducing autoantibodies. Future efforts will be focused on the remaining challenges in disease‐specific skin modeling, which may include improvement of the structural complexity by incorporating dermal layers, vascularization of biofabricated tissues to mimic in vitro drug transportation, fabrication of multidroplet arrays to lead the generation of bulk SOMs, and integration within an air–liquid interface configuration for a full‐thickness skin platform.

## 4 Experimental Section

4.1

4.1.1

##### Cell Reconstruction and Maintenance

A fully assembled GFP‐E‐cad was generated via inserting the GFP‐E‐cad cDNA into the LZBob‐neo‐vector, which is a modified LZRS‐ms‐neo‐vector with multiple cloning sites for increasing cDNA fragment.^[^ ^50^ ^]^ The constructs were transfected into phoenix 293 cells for packaging and amplifying. Phoenix 293 cells were then cultured in a medium which was prepared by Dulbecco's Modified Eagle's Medium (DMEM) (11 965 092, ThermoFisher Scientific) supplemented with 10% fetal bovine serum, 1% penicillin, and 1% GlutaMAX, for more than 2 days. Viral conditioned culture medium was collected and filtered with a 0.45 μm syringe filter. HaCaT cells were infected by culturing in the viral conditioned medium with 4 μg mL^−1^ polybrene (28 728‐55‐4, Sigma) for 7 h at 32 °C. After that, infected HaCaT cells were selected by low‐calcium medium with 500 μg mL^−1^ G418 (Geneticin) until cells were healthy with stable proliferation. Low‐calcium medium was made by replacing DMEM with DMEM with no calcium (21 068 028, ThermoFisher Scientific). To maintain the undifferentiated state, HaCaT cells were cultured in low‐calcium medium at 37 °C supplied with 5% CO_2_ to ≈70% confluency. Then, cells were harvested and resuspended to a concentration of 5 × 10^6^ cells mL^−1^.

##### 3D Biofabrication of Epidermal Models

A typical bioink of the supporting fibrin matrix consisted of 10 mg mL^−1^ fibrinogen (341 576, Millipore), 0.025 mg mL^−1^ aprotinin (A4529, Millipore), and 10% v/v glycerol (G2025, Millipore) in the low‐calcium culture medium. The ink solution was freshly prepared before each fabrication. 1 unit mL^−1^ thrombin was added to crosslink the matrix. The supporting matrix was first printed or cast to each well of a glass‐bottom well plate. Two 1.5 μL hemispherical fibrin gels seeded with 1.5 × 10^3^ GFP‐E‐cad‐HaCaT cells as cell sources were sequentially printed onto the supporting matrix with a controllable distance between 0.8 and 1 mm. The bioprinting process was conducted using a custom‐built 3D bioprinter as reported in our previous studies.^[^ ^34^, ^35^, ^36^ ^]^ The printed samples were cultured for 4 days before imaging, which allowed cells to adapt to the hydrogel matrices. 25 μg mL^−1^ aprotinin was added to the culture medium to stabilize the fibrin matrix,^[^ ^30^ ^]^ and the culture medium was changed with a 4 day interval.

##### Anti‐Dsg3 Antibody Treatment

First, the calcium concentration in culture media for both the control and the AK23‐treated groups was increased to 1.8 mM to induce the formation of Ca‐dependent intercellular adhesions. After culturing overnight in the high‐calcium culture medium, 2 μg mL^−1^ AK23 antibody was added to the samples in the treatment group for another 24 h.

##### Immunostaining

Samples were first washed with DPBS and fixed with 4% paraformaldehyde for 45 min. Then, 0.1% Triton X‐100 was used to permeabilize the samples for 1 h at room temperature. A block solution was prepared using 1% BSA and 22.52 mg mL^−1^ glycine in DPBST (DPBS + 0.1% Tween 20). Primary antibodies were introduced after the samples were blocked for 1 h. After incubating with primary antibodies at ambient temperature for 2 h, the samples were treated with secondary antibodies in 1% BSA solution overnight at 4 °C. Before imaging, samples were also counterstained with DAPI for 2 h. It should be noted that samples were washed at least three times after each step described earlier. The details of primary and secondary antibodies are listed in Table S1, Supporting Information.

##### Imaging Acquisition and Processing

Tissue constructs were imaged using a confocal microscope (LSM800, Zeiss) equipped with an incubation chamber for time‐lapsed observation. Spectral lasers with wavelengths of 405, 488, 561, and 633 nm were used for scanning of the four fluorescent channels. Stitch and *Z*‐stack were performed in Zen Blue software that was associated with the microscope. ImageJ was used to generate composite microscopy images by combining fluorescent channels, maximum orthogonal (*XY*) projection, and 3D rendering and visualization. To minimize the effect of background noise, background subtraction was performed on all raw images before the analysis of fluorescent intensities. All related parameters, including image size, laser power, master gain, and objective pinhole diameter, were optimized for each dye and kept consistent between groups.

##### Analysis of Cell Migration and Proliferation

Multitiles of *Z*‐stacked images of 3D tissue constructs were acquired and stitched. A maximum orthogonal (*XY*) projection of each sample was used for the fluorescent analysis that was conducted with ImageJ. The leading cell that was the furthest distance from the initial boundary of the cell source droplet was tracked at each recording time point. Meanwhile, the summed fluorescence intensity of GFP‐tagged cells was obtained to demonstrate proliferation of HaCaT cells.

##### Fluorescence Intensity Analysis

Fluorescence images were analyzed with ImageJ using the “Analyze Particles” function for each fluorescent channel. For K5 and K10, total particle area and particle area percentage were obtained. For DAPI, average particle area and particle number were calculated to represent average nucleus area and cell population.

##### Dsg3 Mean Intensity and Full Width at Half Maximum Analysis

The analysis was performed by our customized MATLAB scripts. Distribution curves as shown in Figure S8, Supporting Information were plotted by the Dsg3 fluorescent intensity along the line perpendicular to the cell–cell junction. Specifically, a reference line was first drawn bridging the nuclei of two adjacent cells. Dsg3 fluorescent intensities along the reference line were recorded. Based on the microscope camera setting, the pixel number could be converted to relative distance as 0.198 μm pixel^−1^, as shown in Figure 4b,c. Then, the FWHM was calculated as depicted in Figure S8b, Supporting Information to quantify the mean width of a cell–cell junction. Background noise was subtracted based on the average detected intensity of first five pixels at each end of the reference line for the calculation of main peak intensity (*I*_max_−*I*_b_). The maximum (*X*_max_) and minimum (*X*_min_) pixel numbers corresponding to half of each main peak intensity (*I*_half max_) were located. FWHM was finally calculated as the difference of *X*_max_ and *X*_min_. The mean intensity of a single fluorescent image was calculated from the average intensity of the effective pixels. The effective pixel was defined as the pixel with a detectable intensity. Data that was shown in Figure 4d,e were normalized by the untreated control group.

##### Statistical Analysis

Statistical data was analyzed using Origin (data analysis and plotting software). All data of plots and bar charts were presented as quantitative values, shown as mean ± standard deviation, from *n* ≥ 3 independent samples per group of experiments, as stated in the figure captions. Quantile–quantile (Q–Q) plot was used for the normality test. Differences between control and treatment groups were analyzed using unpaired Student's *t*‐test and Mean–Whitney *U*‐test if normality was not met. A *p*‐value of less than 0.05 was considered statistically significant.

## Conflict of Interest

The authors declare no conflict of interest.

## Supporting information

Supplementary Material

## Data Availability

The data that support the findings of this study are available from the corresponding authors upon reasonable request.
