# Supplementary material for: Spatially Guided Construction of Multilayered Epidermal Models Recapturing Structural Hierarchy and Cell–Cell Junctions
Source: Small Sci. 2022 Sep 14;2(11):2200051. doi: 10.1002/smsc.202200051 (PMC9799093; doi:10.1002/smsc.202200051)
Supplement: Supplementary file 1 — Supplementary Material [file SMSC-2-2200051-s001.zip › Spatially Guided Construction of Epidermal Models-R1-SI.pdf]

Supporting Information

Spatially Guided Construction of Multilayered Epidermal Models Recapturing Structural Hierarchy and Cell-Cell Junctions

Haiwei Zhai, Xiaowei Jin, Grayson Minnick, Jordan Rosenbohm, Mohammed Abdul Haleem Hafiz, Ruiguo Yang, and Fanben Meng**

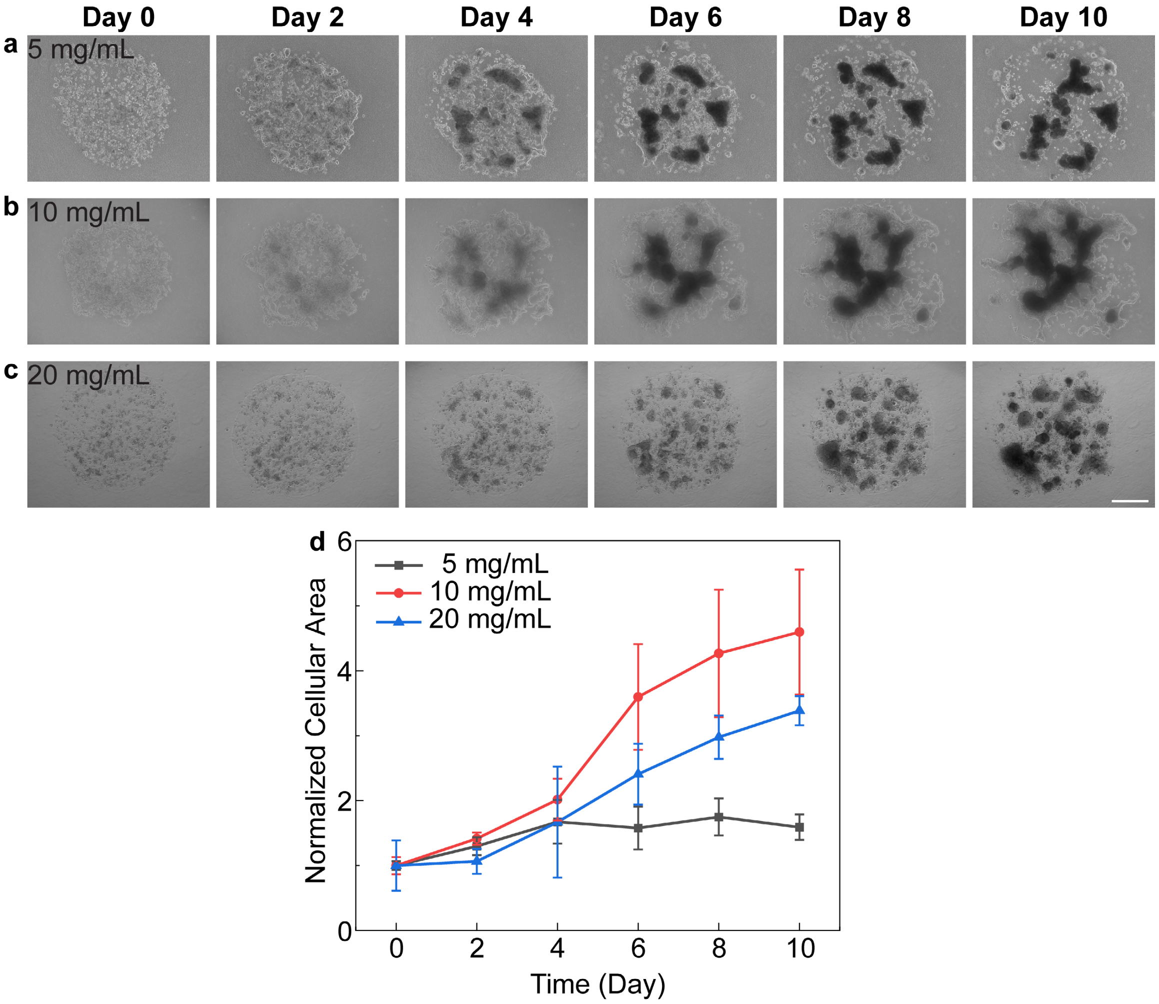

Figure S1. Influence of the fibrin concentration on 3D cultured keratinocytes. Time-lapsed brightfield images of keratinocytes (1 x 10^6 cells/mL) encapsulated in fibrin gel matrices with concentrations of a) 5 mg/mL, b) 10 mg/mL, and c) 20 mg/mL over a 10-day period. Day 0 was defined four days after fabrication. d) Plots of cellular area of keratinocytes and

keratinocyte aggregates within fibrin matrices (Black: 5 mg/mL, Red: 10 mg/mL, and Blue: 20 mg/mL) *vs* time (normalized by the mean of Day 0 of each group, mean ± s.d., n = 5), demonstrating expansion of keratinocytes through the 3D matrices, which is achieved by cell proliferation and migration. 10 mg/mL was selected to maintain the 3D microenvironments for keratinocytes in this study based on the observation of the most rapid cell expansion among three gel concentrations. Scale bar: 500 μm.

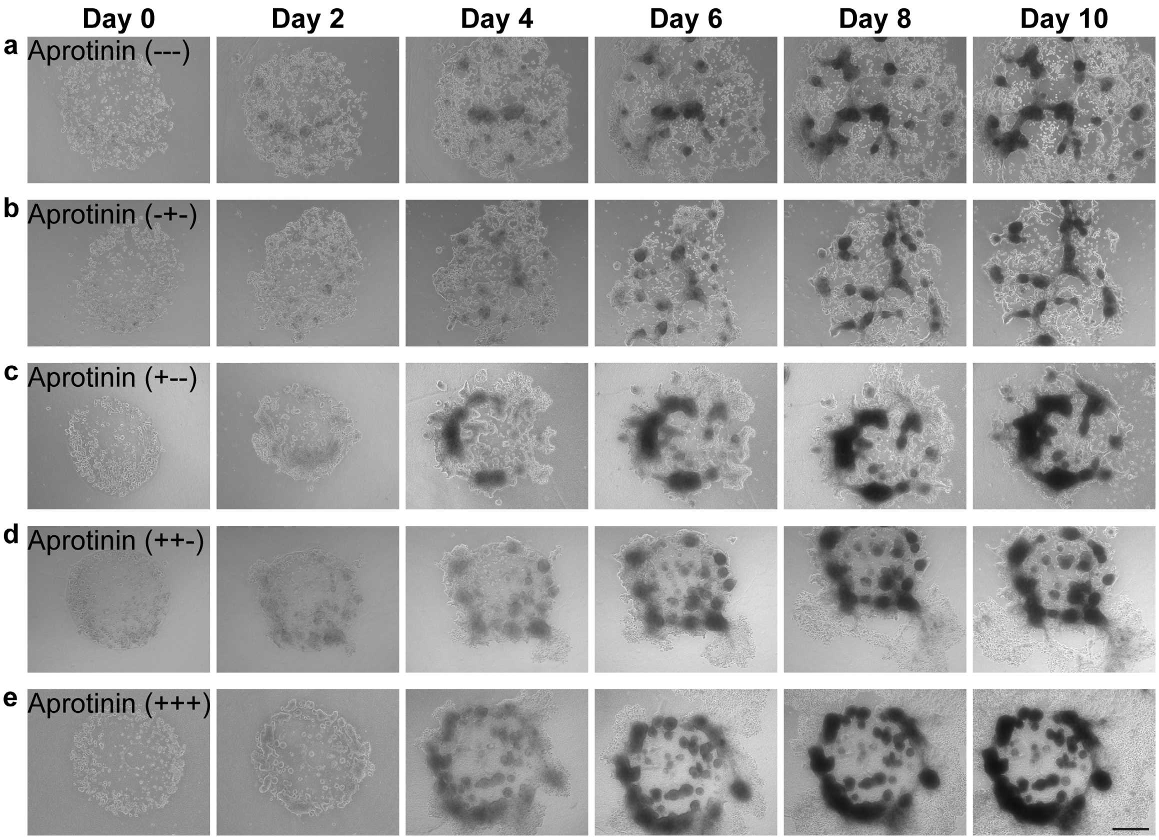

Figure S2. Further tuning fibrin gel stability with addition of aprotinin. Time-lapse brightfield images of keratinocytes encapsulated in 10 mg/ml fibrin gel matrices a) without aprotinin (---) and with 25 μg/mL aprotinin treatments in b) only the cell-laden droplet (-+-), c) only the surrounding matrix (+--), d) both the droplet and the surrounding matrix (++-), and e) the culture media (+++). To promote collective migration of keratinocytes in the surrounding fibrin matrix, option e) was used in this study. Scale bar: 500 μm.

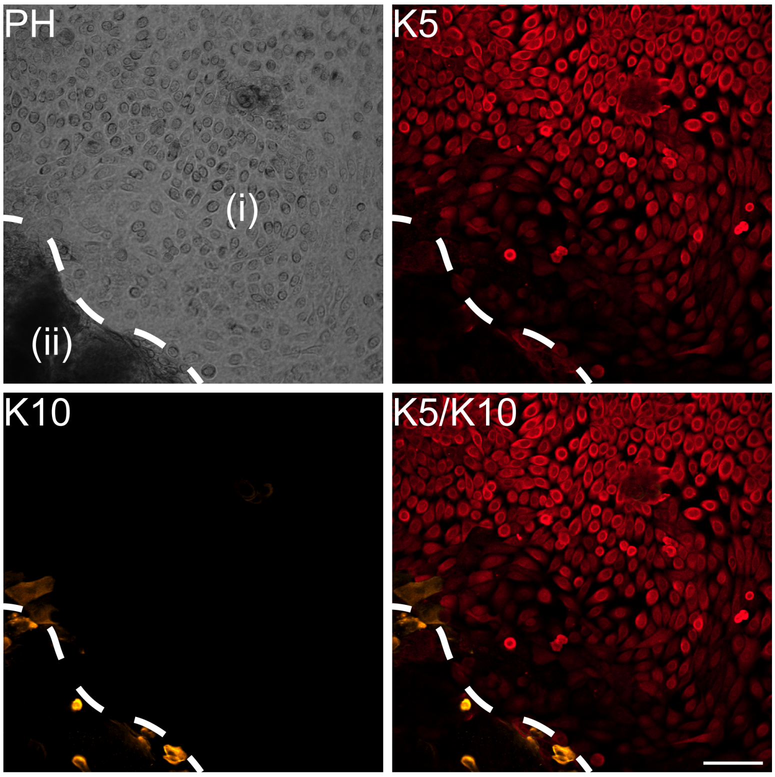

Figure S3. Collective migration of keratinocytes. Keratinocytes proliferated and collectively migrated in the surrounding matrix, Zone (i) from the cell source droplet, Zone (ii), as shown in the brightfield image and fluorescence images with K5 (red, marker for highly proliferative basal cells) and K10 (orange, marker for differentiated suprabasal cells) staining. The keratinocyte differentiation was mainly observed in Zone (II). Scale bar: 100 µm.

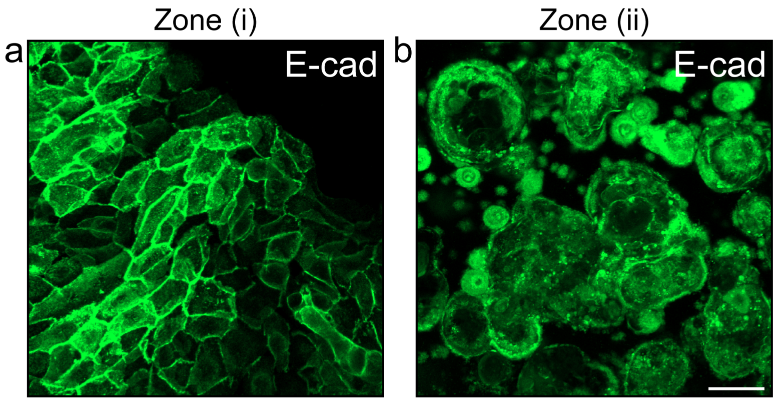

Figure S4. Spatial patterns of 3D-cultured keratinocytes. Representative fluorescence images of GFP-E-cad-HaCaT showing the spatial arrangement of the epidermal cells that a) migrated out of and b) aggregates within the cell source droplet. The distinctive distribution of E-cad that indicated spatial patterns of keratinocytes was observed in different sample regions. Scale bar: 50 µm

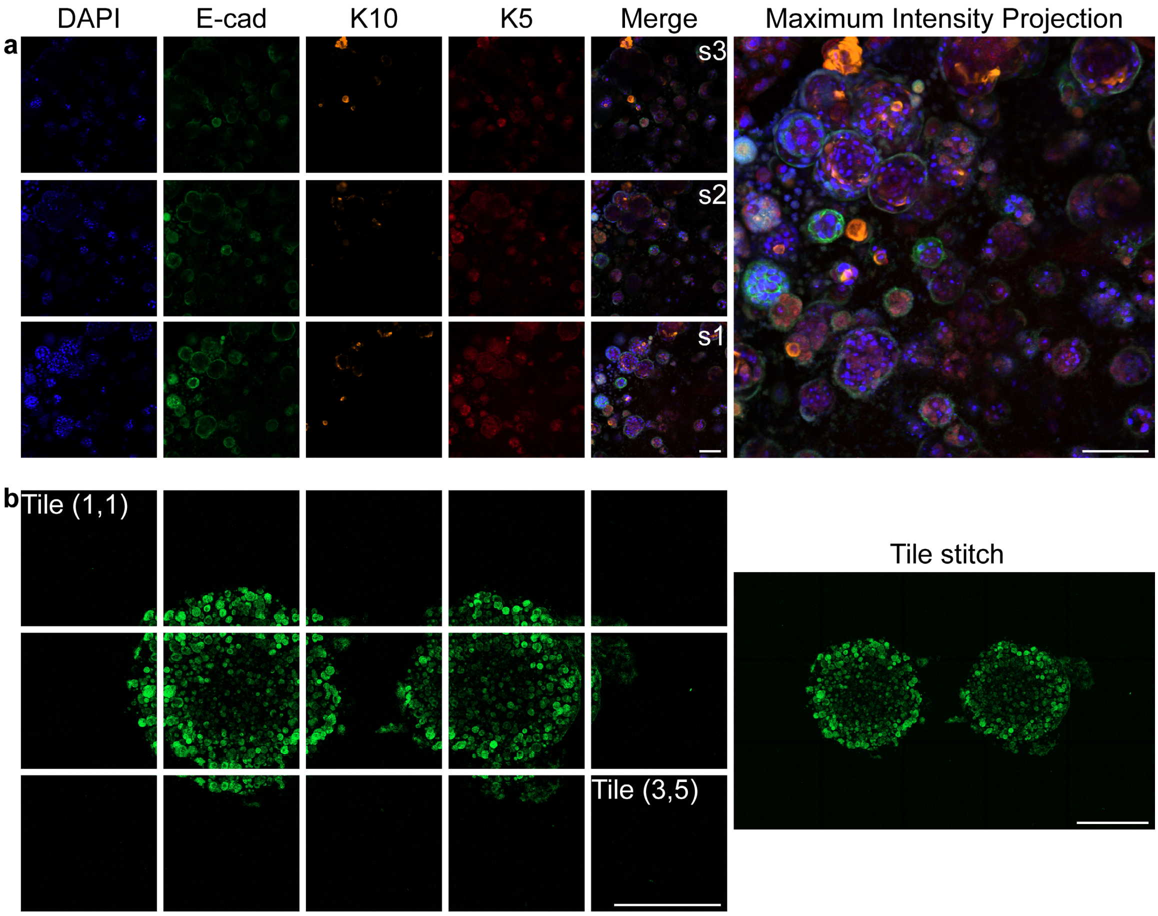

Figure S5. Projection and stitch of confocal images. a) A series of fluorescence images showing the Z-stack of three slices from a vertical multi-channel scan with a Z-step size of 30.02 µm. Scale bar: 100 µm. b) A series of green fluorescence images (tiles for the panoramic images of Figure 2b Day 4) showing the stitch of fifteen tiles with with 10% overlap. Scale bar: 1000 µm.

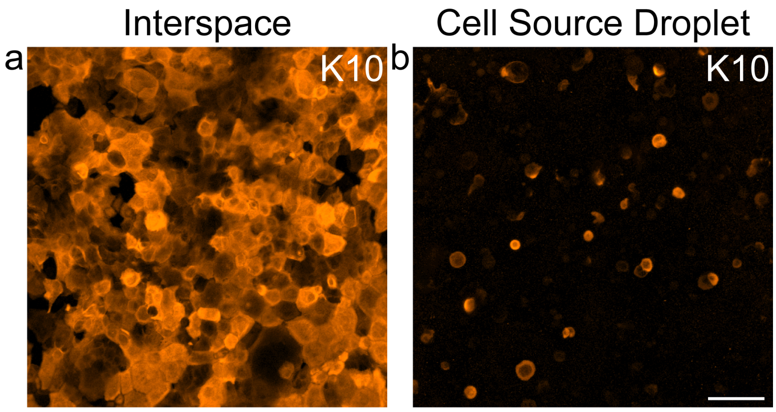

Figure S6. K10 expression in the biofabricated tissue constructs with a pair of cell-laden droplets as geographical cues. Fluorescence images showing K10 expression of keratinocytes located a) in the interspace between two cell source droplets and b) within a cell source droplet. Compared with single-droplet constructs (Figure 1f), the K10 expression within droplets showed a similar pattern while cells that migrated out of droplets significantly enhanced the expression of the differentiation marker in the interspace with the presence of the geographical cue. Scale bar: 100 µm

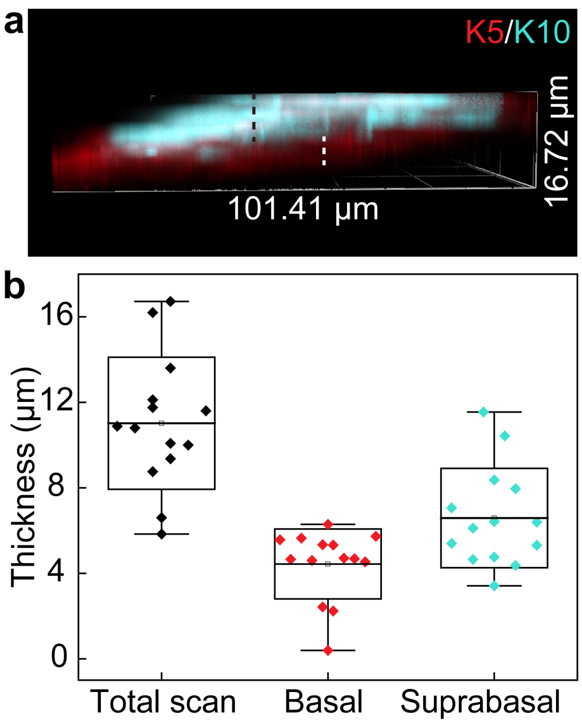

Figure S7. Stratification of SOMs. a) a 3D reconstructed confocal image showing the lateral view of a typical SOM. Scan area: 101.41 µm × 101.41 µm × 16.72 µm. K10 was recolored from orange to turquoise for a better contrast displaying the stratified epidermal tissue. b) Box bar charts showing the measured layer thickness of the total vertical scan, basal and suprabasal layers, respectively. (box: mean ± s.d., error bar: min – max, n: 14 representative regions of three independent samples)

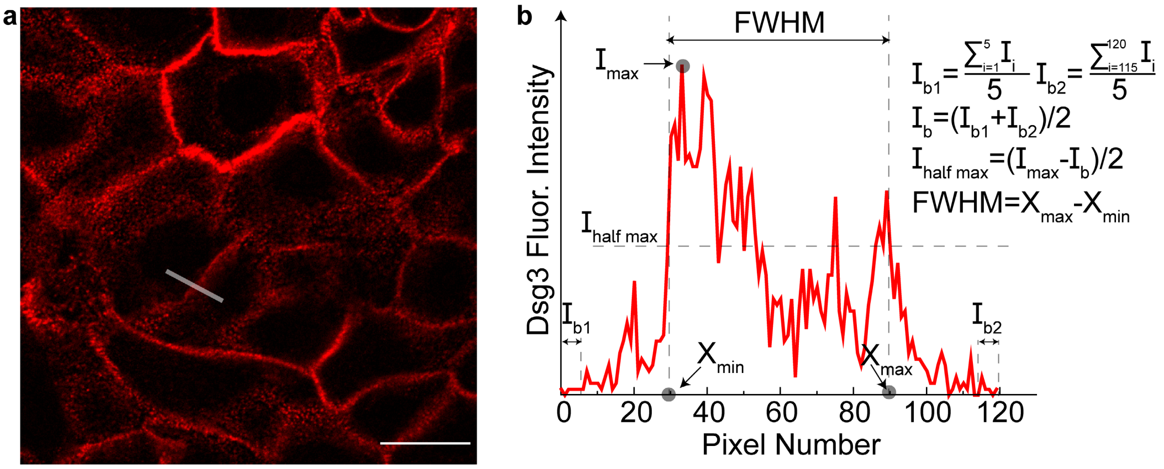

Figure S8. Illustration of full width of half max (FWHM) quantification. a) Red fluorescence image of Dsg3-stained keratinocytes indicating the desmosomal disassembly after a 24-hour treatment of AK23 mAb (2 µg/mL). The white reference line bridges the nuclei of two adjacent cells at a junction site. Scale bar: 20 µm. b) Plots of Dsg3 fluorescence intensity *vs* pixel number along the reference line. I_{max}: Absolute peak intensity; I_b: Background noise that is defined as the average detected intensity of first 5 pixels at each end of the reference line (I_{b1} and I_{b2}); I_{max} - I_b: Relative peak intensity; $I_{half\ max}$: Half of the relative peak intensity; X_{max}: Maximum pixel number where $I_{half\ max}$ is reached; X_{min}: Minimum pixel number where $I_{half\ max}$ is reached; FWHM: X_{max} - X_{min}.

Table S1. Summary of immunostaining reagents

Number	Antibody or dye	Host Species & Reactivity	Manufacturer	Catalog #	Dilution Ratio
1	Anti-Cytokeratin 5 antibody	Mouse anti-human	Thermo Fisher Scientific	MA5-12596	1:250
2	Anti-Cytokeratin 10 antibody	Rabbit anti-human	Abcam	ab76318	1:100
3	Anti-Desmoglein 3/PVA antibody	Mouse anti-human	Abcam	ab231309	1:100
4	Alexa Fluor™ 555	Goat anti-rabbit	Thermo Fisher Scientific	A21430	1:100
5	Alexa Fluor Plus 647	Goat anti-mouse	Thermo Fisher Scientific	A32728	1:100
6	DAPI	DNA	Thermo Fisher Scientific	62247	1:500

Movie Captions

Movie S1. Hierarchical structure of a keratinocyte self-organized multilayer. The movie shows confocal images of keratinocytes in a self-organized multilayer obtained from a multichannel scan with a volume of 1760 µm × 894.09 µm × 50 µm, demonstrating differentiation statuses of keratinocytes located at each layer. 4 channels include K5 (red), K10 (orange, differentiation marker), E-cadherin (green), and DAPI (blue).

Movie S2. Spatial arrangement of keratinocytes in the created interspace. The movie shows the 3D reconstruction of confocal images of DAPI-stained keratinocytes, demonstrating the multilayered distribution and different orientations of keratinocytes. The 3D image was obtained from 5 slices in a vertical scan of 16.2 µm with a scan area of 101.41 µm × 101.41 µm at each slice.

Movie S3. Vertical basal-to-suprabasal transition of multilayered keratinocytes. The movie shows a bottom-up confocal scan of a typical SOM including five slices of keratinocyte layers with a step size of 2.19 µm. The basal-to-suprabasal differentiation of keratinocytes is demonstrated by gradually increased expression of K10 (orange, differentiation marker) and decreased population density observed through DAPI staining (blue).
